# Supplementary material for: Mathematical modelling of mechanotransduction via RhoA signalling pathways
Source: PLoS Comput Biol. 2025 Jul 31;21(7):e1013305. doi: 10.1371/journal.pcbi.1013305 (PMC12327677; doi:10.1371/journal.pcbi.1013305)
Supplement: S1 Appendix — The appendix contains a comparison of the reduced model and the full model of [18] in Sect A.1, temporal statistics in Sect A.2, the numerical scheme in Sect A.3, the conversion from μM to #/μm2 in Sect A.4, simulations for the model with nucleus in Sect A.5, a parameter sensitivity analysis in Sect A.6, and simulations for viscoelastic model in Sect A.7. (PDF) [file pcbi.1013305.s001.pdf]

S1 Appendix

A.1 Comparison of the reduced model and the full model of [1]

We verify the reduced model (1) captures the results obtained in [1] for the full model for the RhoA signalling pathway. In numerical simulations of model (1) we use the same parameter values as in [1], except for the diffusion coefficients D_1 and D_2 for activated and deactivated FAK. It is suggested in the literature that $D_1 = D_2 = 4\ \mu\text{m}^2/s$ [2], but [1] uses $10\ \mu\text{m}^2/s$ due to computational issues. Thus in our numerical simulations we consider both diffusion coefficients.

$\phi_d^0 = 0.7\mu\text{mol/dm}^3$	$C = 3.25$ kPa	$D_1 = 4$ or $10\ \mu\text{m}^2/\text{s}$	$k_2 = 0.015\ \text{s}^{-1}$
$\phi_a^0 = 0.3\mu\text{mol/dm}^3$	$\gamma = 8.8068\ \text{dm}^3/\mu\text{mol}$	$D_2 = 4$ or $10\ \mu\text{m}^2/\text{s}$	$k_3 = 0.379\ \text{s}^{-1}$
$\rho_a^0 = 33.6\ \#/\mu\text{m}^2$	$n = 5$	$D_3 = 0.3\ \mu\text{m}^2/\text{s}$	$k_4 = 0.625\ \text{s}^{-1}$
$\approx 6 \cdot 10^{-7}\ \mu\text{mol/dm}^2$	$E = 0.1, 5.7, 7 \cdot 10^6$ kPa	$k_1 = 0.035\ \text{s}^{-1}$	$k_5 = 0.0168\ \text{s}^{-1}$
$\rho_d^0 = 1\ \mu\text{mol/dm}^3$	$\lvert Y \rvert = 1193\ \mu\text{m}^3$	$\lvert\Gamma\rvert = 1020\ \mu\text{m}^2$	

Table A1. Parameter values for model (1).

The model (1) is implemented in FEniCS [3], using a Finite Element Method for discretization in space and IMEX time-stepping method to discretize in time, see Section A.3 for more details. Considering domain $Y \subset \mathbb{R}^3$, denoting the cytoplasm, and $\Gamma = \partial Y$, defining the cell membrane, and times interval $(0, T)$, with $T = 100$ s, for the space discretisation we choose meshsize $h = 2.94$ and time step $\Delta t = 0.5$ for the backwards Euler discretisation in time. For our domain we have $n_r = |Y|/|\Gamma| = 1.17\ \mu$m. We consider three different stimuli, similar to [1], (i) the '2D stimulus', where the substrate stiffness is only applied to the bottom of the cell and any reaction terms of RhoA are nonzero only at the bottom of the cell, (ii) the '2xD stimulus', where the substrate stiffness is only applied to the bottom of the cell but the reaction terms of RhoA are nonzero on the whole cell membrane, and (iii) the '3D stimulus' where the cell is embedded in an agar (substrate) and the impact of the substrate stiffness on the signalling processes is considered on the whole cell membrane.

Comparing the simulation results for reduced model (1) in Figs A1 and A2 to the results presented in [1, Fig 3] for the full model, the dynamics of FAK and RhoA are almost identical qualitatively. Similar to the results in [1, Fig 3], the highest concentration of both ϕ_a and ρ_a is at the edges of the cell. Also, there is threshold value of $E \approx 1$kPa, below which the concentrations of ϕ_a and ρ_a stay close to the initial values and then reaches high steady states values, similar for both $E = 5.7$kPa and $E = 7$GPa. Quantitatively, the values for ϕ_a are also close to the one reported in [1, Fig 3]. This suggests that the reduction of the model as well as considering the whole cell domain without excluding a nucleus does not have significant effect on the dynamics of FAK. However, for ρ_a we obtain slightly lower concentration, where the maximum concentration in our results is $420\#/\mu m^2$ and the maximum concentration in [1, Fig.3] is $593\#/\mu m^2$. This difference could be related to the model reduction and approximation for the deactivated RhoA. The differences in the conversion from $\mu\text{mol}/dm^2$ to $\#/\mu m^2$ using our approach, see Section A.4 for details, and the approach of [1] may also contribute.

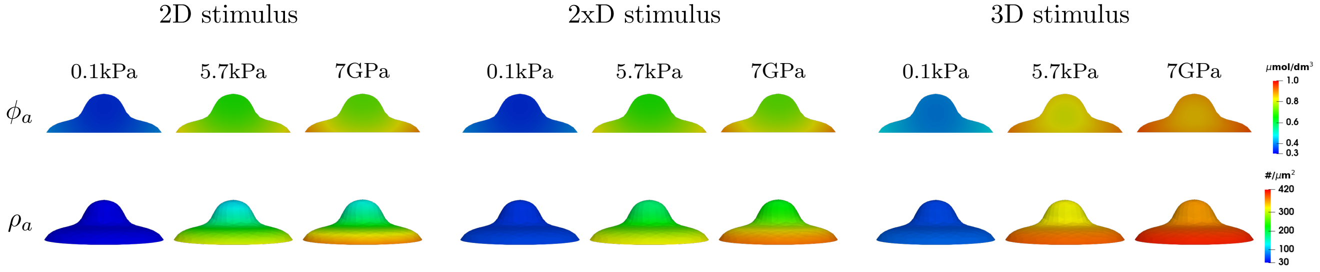

Fig A1. Numerical simulation results showing ϕ_a and ρ_a for reduced model (1) at steady state for $T = 100$ s. Parameter values as in Table A1 and $D_1 = D_2 = 4\ \mu\text{m}^2/\text{s}$.

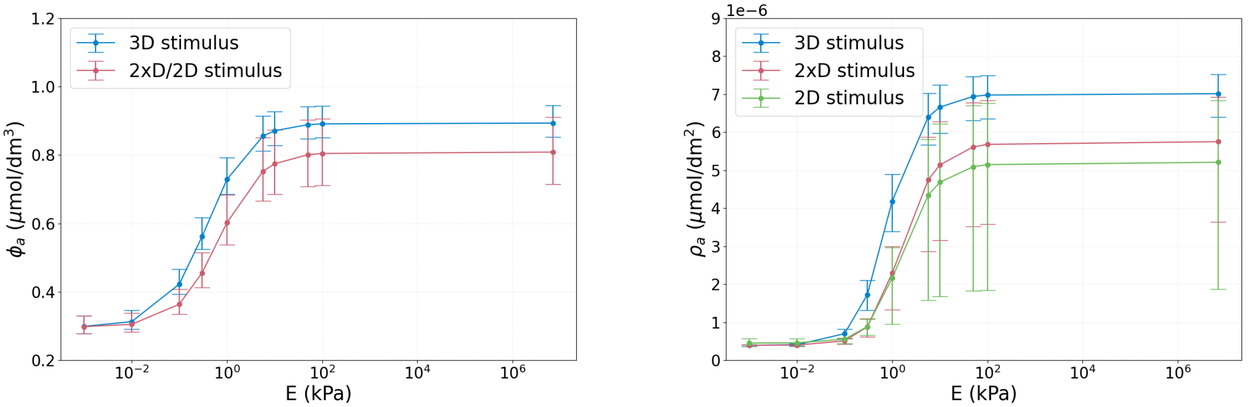

Fig A2. Results showing the effect of substrate stiffness E on ϕ_a and ρ_a for the reduced model (1) at $T = 100$ s by which time the results are at a steady state. Parameter values as in Table A1 with $D_1 = D_2 = 4\ \mu\text{m}^2/\text{s}$.

Simulation results for the reduced model with diffusion coefficients $D_1 = D_2 = 10\mu\text{m}^2/\text{s}$ are presented in Figs A3 and A4. Comparing Figs A2 and A4, we see that the averaged over space dynamics are very similar, but numerical simulation results for a lower diffusion coefficient show lower minimum and higher maximum concentrations, which can be explained by the fact that slower diffusion of ϕ_a causes stronger heterogeneity across the cell domain. In Figs A1 and A3, we observe the same dynamics for both diffusion coefficients, where the maximum concentration is at the edges of the cell and the minimum concentration is in the middle. Since the dynamics of ρ_a depends on ϕ_a, similar results are obtained for ρ_a.

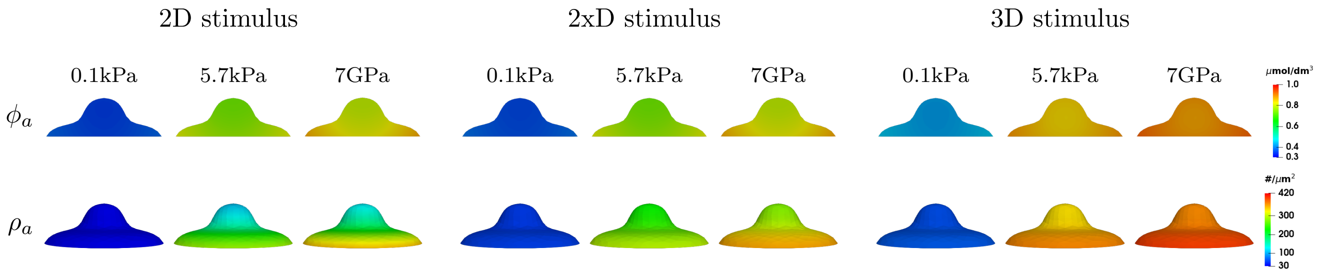

Fig A3. Numerical simulation results showing ϕ_a and ρ_a for reduced model (1) at $T = 100$ s by which time the results are at a steady state. Parameter values as in Table A1 with $D_1 = D_2 = 10\mu\text{m}/s$.

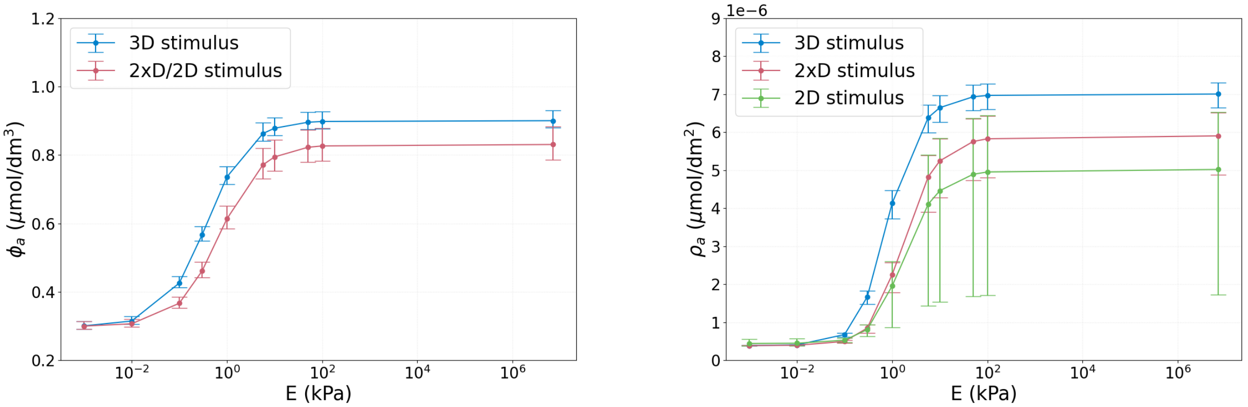

Fig A4. Results showing effect of substrate stiffness E on ϕ_a and ρ_a for the reduced model (1) at $T = 100$ s by which time the results are at a steady state. Parameter values as in Table A1 with $D_1 = D_2 = 10\mu\text{m}/s$.

A.2 Temporal statistics

Fig A5 shows the evolution of the mean of $f(\phi_a)$, $\mathrm{div}(u)$, ϕ_a and ρ_a over time for different couplings and parameters for the axisymmetric cell shape.

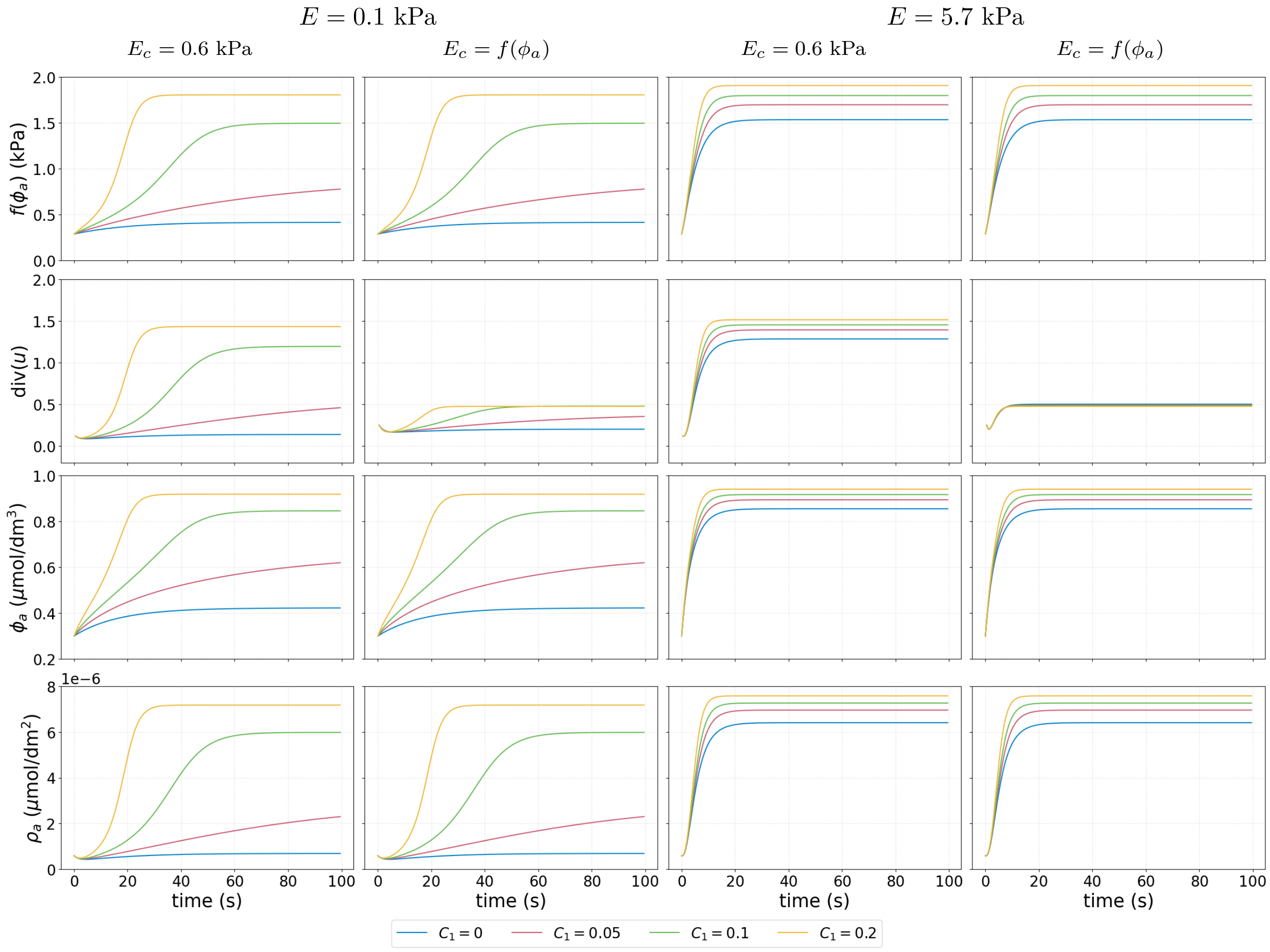

Fig A5. Simulations results showing the mean of $f(\phi_a)$, $\mathrm{div}(u)$, ϕ_a and ρ_a as functions of time, in the case of model (3), (4) and (6), axisymmetric shape and 3D stimulus, for different couplings, four different values for C_1, and two different values for the substrate stiffness E. All other parameter values as in Table 1. The corresponding results can be found in Fig 7.

A.3 Numerical Scheme

For numerical simulations of model (3)-(6) we use FEM for discretization in space and backward Euler for discretization in time, implemented in FEniCS [3]. Consider the space

$$\mathcal{W}(Y) = \{u \in H^1(Y) : \int_Y u_i \,\mathrm{d}x = 0, \int_Y \left(\partial_{x_j} u_i - \partial_{x_i} u_j\right) \mathrm{d}x = 0 \quad \text{for} \;\; i,j = 1,2,3\}, \tag{A1}$$

such that $\mathcal{W}(Y) \cap \mathcal{R}(Y) = 0$ with $\mathcal{R}(Y)$ the space of rigid motions. Then, the weak solution of the model (3)-(6) is given by $(\phi_d, \phi_a) \in L^2(0,T; H^1(Y))$, $\rho_a \in L^2(0,T; H^1(\Gamma))$, with $(\partial_t \phi_d, \partial_t \phi_a) \in L^2(0,T; H^1(Y)')$ and $\partial_t \rho_a \in L^2(0,T; H^1(\Gamma)')$, and $u \in L^2(0,T; \mathcal{W}(Y))$ satisfying

$$\begin{aligned}
&\langle \partial_t \phi_d, \psi \rangle_{(H^1)',T} + \langle D_1 \nabla \phi_d, \nabla \psi \rangle_{Y_T} + \langle C_1 \,\mathrm{tr}(\sigma(u))_+ \phi_d, \psi \rangle_{Y_T} + \langle n_r \tilde{k}_3 \phi_d, \psi \rangle_{\Gamma_T} = \langle k_1 \phi_a, \psi \rangle_{Y_T}, \\
&\langle \partial_t \phi_a, \varphi \rangle_{(H^1)',T} + \langle D_2 \nabla \phi_a, \nabla \varphi \rangle_{Y_T} + \langle k_1 \phi_a, \varphi \rangle_{Y_T} = \langle C_1 \,\mathrm{tr}(\sigma(u))_+ \phi_d, \varphi \rangle_{Y_T} + \langle n_r \tilde{k}_3 \phi_d, \psi \rangle_{\Gamma_T}, \\
&\langle \partial_t \rho_a, w \rangle_{(H^1)',T} + \langle D_3 \nabla_\Gamma \rho_a, \nabla_\Gamma w \rangle_{\Gamma_T} + \langle \tilde{k}_4(\phi_a) \rho_a, w \rangle_{\Gamma_T} = \langle n_r \tilde{k}_5(\phi_a), w \rangle_{\Gamma_T}, \\
&\langle E(\phi_a) \epsilon(u), \epsilon(v) \rangle_{Y_T} = \langle k_6 \mathbb{P}(\rho_a \nu), v \rangle_{\Gamma_T},
\end{aligned} \tag{A2}$$

for all $\psi, \varphi \in L^2\left(0,T;H^1(Y)\right)$, $w \in L^2\left(0,T;H^1(\Gamma)\right)$, and $v \in L^2(0,T;H^1(Y))$, with initial conditions satisfied in the L^2-sense, and where $\tilde{k}_3 = k_2 + k_3\frac{E}{C+E}$, $\tilde{k}_4(\phi_a) = k_4 + k_5((\gamma\phi_a)^n + 1)$ and $\tilde{k}_5(\phi_a) = k_5((\gamma\phi_a)^n + 1)\frac{M_\rho}{|Y|}$. Here $\langle \phi, \psi \rangle_{(H^1)',T}$ denotes the dual product between $\phi \in L^2(0,T;H^1(Y))$ and $\psi \in L^2(0,T;H^1(Y)')$ or between $\phi \in L^2(0,T;H^1(\Gamma))$ and $\psi \in L^2(0,T;H^1(\Gamma)')$ and

$$\langle \phi, \psi \rangle_{Y_T} = \int_0^T \int_Y \phi\psi \,\mathrm{d}x\,\mathrm{d}t\,, \quad \langle \phi_1, \psi_1 \rangle_{\Gamma_T} = \int_0^T \int_\Gamma \phi_1\psi_1 \,\mathrm{d}x\,\mathrm{d}t\,, \qquad \text{where } 1/p_1 + 1/p_2 = 1,\;\; 1/q_1 + 1/q_2 = 1,$$

for $\phi \in L^{p_1}(0,T;L^{q_1}(Y))$, $\psi \in L^{p_2}(0,T;L^{q_2}(Y))$, $\phi_1 \in L^{p_1}(0,T;L^{q_1}(\Gamma))$, and $\psi_1 \in L^{p_2}(0,T;L^{q_2}(\Gamma))$. The discretization of the domain is given by the polyhedral approximation of Y such that Y_h is the union of finitely many tetrahedrons in $\mathbb{R}^3$, and S_h is the set of these tetrahedrons K, such that

$$Y_h = \bigcup_{K \in S_h} K.$$

Then the surface Γ is approximated by Γ_h such that $\Gamma_h = \partial Y_h$. The mesh size is defined by the maximum diameter of a simplex $h = \max\{h_Y, h_\Gamma\}$, where $h_Y = \max_{K \in S_h} h_Y(K)$ and $h_\Gamma = \max_{R = K \cap \Gamma_h \neq 0, K \in S_h} h_\Gamma(R)$ with $h_Y(K)$ being the diameter of a tetrahedron and $h_\Gamma(R)$ the diameter of a triangle on the surface.

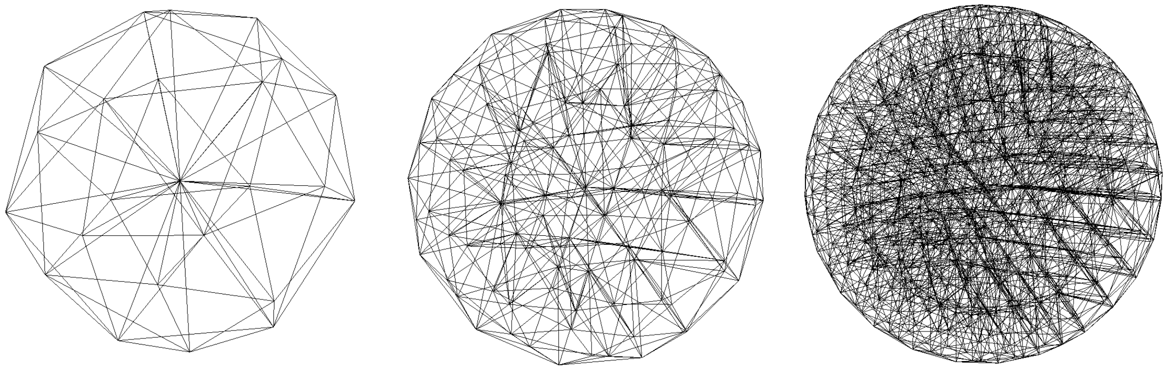

Fig A6. Tetrahedral approximations of a unit sphere with, from left to right, decreasing values of h, created with Gmsh [4].

The bulk and surface finite element spaces are given by

$$\begin{aligned}
\mathbb{V}_{h,Y} &= \left\{\Psi \in C(Y_h) : \Psi|_K \text{ is linear affine for each } K \in S_h\right\},\\
\mathbb{V}_{h,\Gamma} &= \left\{\Psi \in C(\Gamma_h) : \Psi|_R \text{ is linear affine for each } K \in S_h \text{ with } R = K \cap \Gamma_h \neq 0\right\},\\
\mathbb{W}_{h,Y} &= \left\{V \in \mathcal{W}(Y_h) \text{ and } V_i \in C(Y_h) : V_i|_K \text{ is linear affine for each } K \in S_h \text{ for } i = 1,2,3\right\},
\end{aligned}$$

where we recall the definition of $\mathcal{W}$ in Eq (A1). The bulk space is spanned by nodal basis functions defined by

$$\chi_j \in \mathbb{V}_{h,Y}, \quad \chi_j(X_k) = \delta_{jk} \quad \text{for } \; j,k = 1,...,J,$$

where δ_{jk} is the Kronecker delta and J is the number of nodes (vertices) $X_j \in Y$ of the tetrahedrons S_h. Then function $\Phi(t) \in \mathbb{V}_{h,Y}$ has the form

$$\Phi(t,x) = \sum_{j=1}^{J} a_j(t)\chi_j(x) \quad \text{ for } \; x \in Y_h,\; t \in (0,T),$$

with real measurable functions a_j. Similarly the surface finite element space is spanned by nodal basis functions

$$\mu_j \in \mathbb{V}_{h,\Gamma}, \quad \mu_j(Z_k) = \delta_{jk} \quad \text{for } \; j,k = 1,...,M,$$

where $Z_j \in \Gamma$, with $j,k = 1,...,M$, are nodes of the triangulated surface such that $Z_k = X_k \bigcap \Gamma_h \neq 0$. Then function $P(t) \in \mathbb{V}_{h,\Gamma}$ has the form

$$P(t,x) = \sum_{j=1}^{M} b_j(t)\mu_j(x) \quad \text{ for } \; x \in \Gamma_h,\; t \in (0,T),$$

with real measurable functions b_j. Thus, the semi-discretized problem corresponding to (3)-(6) reads

$$\begin{aligned}
&\langle\partial_t\Phi_d,\Psi\rangle_{Y_h}+\langle D_1\nabla\Phi_d,\nabla\Psi\rangle_{Y_h}+\left\langle C_1\operatorname{tr}(\sigma(U))_+\Phi_d,\Psi\right\rangle_{Y_h}+\left\langle n_r\tilde{k}_3\Phi_d,\Psi\right\rangle_{\Gamma_h}=\langle k_1\Phi_a,\Psi\rangle_{Y_h},\\
&\langle\partial_t\Phi_a,\Psi\rangle_{Y_h}+\langle D_2\nabla\Phi_a,\nabla\Psi\rangle_{Y_h}+\langle k_1\Phi_a,\Psi\rangle_{Y_h}=\left\langle C_1\operatorname{tr}(\sigma(U))_+\Phi_d,\Psi\right\rangle_{Y_h}+\left\langle n_r\tilde{k}_3\Phi_d,\Psi\right\rangle_{\Gamma_h},\\
&\langle\partial_t P_a,W\rangle_{\Gamma_h}+\langle D_3\nabla_{\Gamma_h}P_a,\nabla_{\Gamma_h}W\rangle_{\Gamma_h}+\left\langle\tilde{k}_4(\Phi_a)P_a,W\right\rangle_{\Gamma_h}=\left\langle n_r\tilde{k}_5(\Phi_a),W\right\rangle_{\Gamma_h},\\
&\langle E(\Phi_a)\epsilon(U),\epsilon(V)\rangle_{Y_h}=\langle k_6\mathbb{P}(P_a\hat{\nu}),V\rangle_{\Gamma_h},
\end{aligned}\tag{A3}$$

for every test function $\Psi\in\mathbb{V}_{h,Y}$, $W\in\mathbb{V}_{h,\Gamma}$ and $V\in\mathbb{W}_{h,Y}$.

To obtain the fully discrete problem we discretize (A3) in time using the backwards Euler method with

$$\partial_t\Phi^n\approx\frac{\Phi^n-\Phi^{n-1}}{\Delta t},$$

where $\Delta t=T/N$, and an IMEX time-stepping method, in which the diffusion terms are treated implicitly and the nonlinear reaction terms are treated explicitly [5]. The discrete system, with the notation $\Phi^n(x)=\Phi(t_n,x)$, reads

$$\begin{aligned}
\left\langle\mathbb{E}(\Phi_a^{n-1})\epsilon(U^n),\epsilon(V)\right\rangle_{Y_h} &= \left\langle k_6\mathbb{P}(P_a^{n-1}\hat{\nu}),V\right\rangle_{\Gamma_h},\\
\Delta t^{-1}\langle\Phi_d^n,\Psi\rangle_{Y_h}+\langle D_1\nabla\Phi_d^n,\nabla\Psi\rangle_{Y_h} &+ \left\langle C_1\operatorname{tr}(\sigma(U^n))_+\Phi_d^n,\Psi\right\rangle_{Y_h}+\left\langle n_r\tilde{k}_3\Phi_d^n,\Psi\right\rangle_{\Gamma_h}\\
&= \Delta t^{-1}\left\langle\Phi_d^{n-1},\Psi\right\rangle_{Y_h}+\left\langle k_1\Phi_a^{n-1},\Psi\right\rangle_{Y_h},\\
\Delta t^{-1}\langle\Phi_a^n,\Psi\rangle_{Y_h}+\langle D_2\nabla\Phi_a^n,\nabla\Psi\rangle_{Y_h} &+ \langle k_1\Phi_a^n,\Psi\rangle_{Y_h}\\
&= \Delta t^{-1}\left\langle\Phi_a^{n-1},\Psi\right\rangle_{Y_h}+\left\langle C_1\operatorname{tr}(\sigma(U^n))_+\Phi_d^n,\Psi\right\rangle_{Y_h}+\left\langle n_r\tilde{k}_3\Phi_d^n,\Psi\right\rangle_{\Gamma_h},\\
\Delta t^{-1}\langle P_a^n,W\rangle_{\Gamma_h}+\langle D_3\nabla_{\Gamma_h}P_a^n,\nabla_{\Gamma_h}W\rangle_{\Gamma_h} &+ \left\langle\tilde{k}_4(\Phi_a^n)P_a^n,W\right\rangle_{\Gamma_h}\\
&= \Delta t^{-1}\left\langle P_a^{n-1},W\right\rangle_{\Gamma_h}+\left\langle n_r\tilde{k}_5(\Phi_a^n),W\right\rangle_{\Gamma_h}.
\end{aligned}\tag{A4}$$

To benchmark the numerical scheme and implementation in FEniCS, we consider Y to be a unit ball and a simplified model

$$\begin{aligned}
-\nabla\cdot\sigma(u)&=f && \text{in } Y,\\
\sigma(u)\cdot\nu&=\mathbb{P}(g\rho) && \text{on } \Gamma,\\
\partial_t\phi-\Delta\phi&=q_1+\operatorname{tr}(\sigma(u))_+ && \text{in } Y,\ t>0,\\
\nabla\phi\cdot\nu&=\rho-\phi && \text{on } \Gamma,\ t>0,\\
\partial_t\rho-\Delta_\Gamma\rho&=q_2-\rho+\phi && \text{on } \Gamma,\ t>0,\\
u(0,x)=u_{ex}(0,x),\quad \phi(0,x)&=\phi_{ex}(0,x) && \text{in } Y,\\
\rho(0,x)&=\rho_{ex}(0,x) && \text{on } \Gamma.
\end{aligned}\tag{A5}$$

The functions f, g, q_1, and q_2 are such that

$$\begin{aligned}
u_{ex}(t,x)&=\left(2x_1^2x_2x_3e^{-4t},-x_1x_2^2x_3e^{-4t},-2x_1x_2x_3^2e^{-4t}\right),\\
\phi_{ex}(t,x)&=\cos(x_1x_2x_3)e^{-4t},\\
\rho_{ex}(t,x)&=\cos(x_1x_2x_3)e^{-4t}-3\sin(x_1x_2x_3)e^{-4t}
\end{aligned}$$

is the exact solution of (A5). Then for the experimental order of convergence

$$\mathrm{EOC}=\frac{\log(e_n/e_{n-1})}{\log(h_n/h_{n-1})},$$

where h_n, for $n=1,2,3,4$, are given in Table (A2) and e_n is the error in the L^2-norm or the H^1-norm, we obtain the second order of convergence in the L^2-norm and first order of convergence in H^1-norm, see Table A3.

0.64009064	0.51659533	0.26133991	0.13143219

Table A2. Four mesh sizes used in the calculation of EOC.

EOC for L^2 norm for ϕ	1.63892969	1.77954013	1.93991995
EOC for H^1 norm for ϕ	1.44823967	1.68924575	1.89210920
EOC for L^2 norm for ρ	1.58078852	1.78119701	1.96179159
EOC for H^1 norm for ρ	1.78659381	1.49338646	1.52956398
EOC for L^2 norm for u	0.92059071	1.74758321	1.87989078
EOC for H^1 norm for u	1.05358979	1.40005535	1.27787725

Table A3. Experimental order of convergence for numerical scheme (A4) considered for model (A5).

A.4 Conversion from μmol/dm^2 to $\#/\mu m^2$

Scott et al. [1] uses μmol/dm^3 for concentrations in the cytoplasm and $\#/\mu m^2$ for concentrations on the plasma membrane, specifically for ρ_a. In the models derived and analysed in this work, we use μmol/dm^3 for concentrations in the cytoplasm and μmol/dm^2 for ρ_a. To be able to use the same initial conditions and to compare the results, we need to find a conversion from $\#/\mu m^2$ to μmol$/dm^2$. We use the fact that the maximum value for ρ_a for large E is $11 \cdot 10^{-16}\mu$mol/μm^2 as given in [1, Fig 2B]. The maximum value in the numerical results for ρ_a for large E is 593#/μm^2 as given in [1, Fig 3C(ii)] and assume these are equivalent. This relation gives the conversion

$$10^{-5}\frac{\mu\text{mol}}{\text{dm}^2} = 10^{-15}\frac{\mu\text{mol}}{\mu\text{m}^2} = \frac{5930}{11}\#/\mu\text{m}^2 = 539.\overline{09}\#/\mu\text{m}^2. \tag{A6}$$

A.5 Simulations with nucleus

To model the inclusion of a nucleus in the cell, we consider the model in Eqs (3) and (6) in $Y \setminus \overline{Y}_{\text{nc}}$, where domain Y_{nc} represents the nucleus. We choose zero flux boundary conditions for ϕ_d and ϕ_a on ∂Y_{nc}. For the mechanics, we choose the interior boundary condition to model the fact that the nucleus is hard to deform

$$\sigma(u)\nu = -\omega u \quad \text{on } \partial Y_{\text{nc}}, \tag{A7}$$

where ω is a positive constant determining the rigidity of the nucleus.

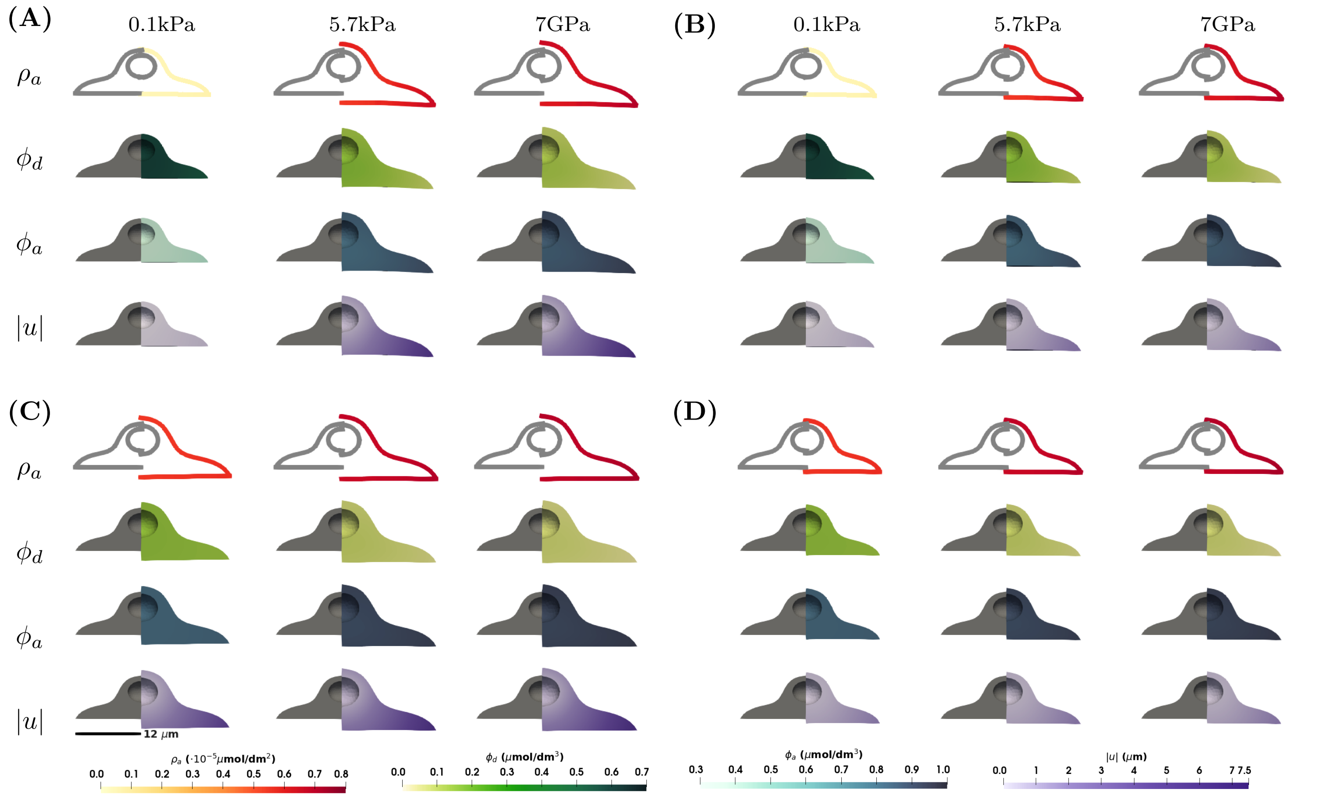

Fig A7. Numerical simulation results showing ρ_a, ϕ_d, ϕ_a and $|u|$ for model (3), (4), (6), and (A7) for the axisymmetric shape with a nucleus and in the case of the 3D stimulus at a steady state at $T = 100$ s. Four different scenarios are considered: **(A)** $C_1 = 0$ (kPa s)$^{-1}$ ($\sigma \not\rightarrow \phi_a$) and $E_c = 0.6$ kPa ($\phi_a \not\rightarrow E_c$); **(B)** $C_1 = 0$ (kPa s)$^{-1}$ ($\sigma \not\rightarrow \phi_a$) and $E_c = f(\phi_a)$ ($\phi_a \rightarrow E_c$); **(C)** $C_1 = 0.1$ (kPa s)$^{-1}$ ($\sigma \rightarrow \phi_a$) and $E_c = 0.6$ kPa ($\phi_a \not\rightarrow E_c$); **(D)** $C_1 = 0.1$ (kPa s)$^{-1}$ ($\sigma \rightarrow \phi_a$) and $E_c = f(\phi_a)$ ($\phi_a \rightarrow E_c$). Within each subfigure, the rows represent ρ_a, ϕ_d, ϕ_a and $|u|$ on a cross-section of the plane $x_1 = 0$ of the axisymmetric cell, and the columns represent $E = 0.1, 5.7, 7 \cdot 10^6$ kPa. Parameter values as in Table 1, and $\omega = 1$. The corresponding results without a nucleus can be found in Fig 7.

A.6 Parameter sensitivity analysis

As we compare and extend upon the model of [1], we have used the same parameter values where possible. For all new values introduced in this work, see Table 1, we provide here a simple parameter sensitivity analysis to explore how different choices of these parameters would affect the results. We choose to focus on the case of the 3D stimulus for the model in Eqs (3)-(4), (6) with force boundary conditions on the whole cell membrane for the axisymmetric cell, where $E_c = f(\phi_a)$ and $C_1 = 0.1$ (kPa s)$^{-1}$ when not otherwise specified, and for $E = 0.1$ kPa, 5.7 kPa, 7 GPa. Parameter values are chosen as in Table 1 and we show summary statistics for a 10% and 20% change in values for each of the following parameters separately: C_1, k_6, k_7, k_8, p and ν_c.

In Fig A8, we see all parameters only have a small effect on the concentrations of ϕ_a and ρ_a with C_1 and k_6 being the most influential when $E = 0.1$ kPa. The parameters k_7, k_8, p and ν_c are part of the equations of linear elasticity and therefore their effect would be through changes in the stress in the reaction term of ϕ_a, which is also determined by C_1. The change in the stress due to the change in these parameters barely changes the results of the signalling molecules. Next to that, we see that the effect is larger for smaller substrate stiffness E, which could be due to the fact that ϕ_a and ρ_a are already close to their maximum values for larger substrate stiffnesses. Fig A8 shows that the effect of C_1 on the change in ρ_a is approximately tripled compared to the change in ϕ_a. This could be because the reaction term including ϕ_a in ρ_a is to the power of 5.

Fig A8 also shows that k_7, k_8, and p have an opposite effect on the cell stiffness E_c and the change in volume div(u), where an increase in these parameters increases the stiffness and decreases div(u). The parameter k_8 has the largest effect on these variables, where a -20% change has a tripling effect of a $+60\%$ change in div(u). Lastly, we see that ν_c only significantly changes div(u). The effect that ν_c, the Poisson ratio, determining the ratio between the transverse and the axial strain, has on the volume change does not seem to induce changes in other variables.

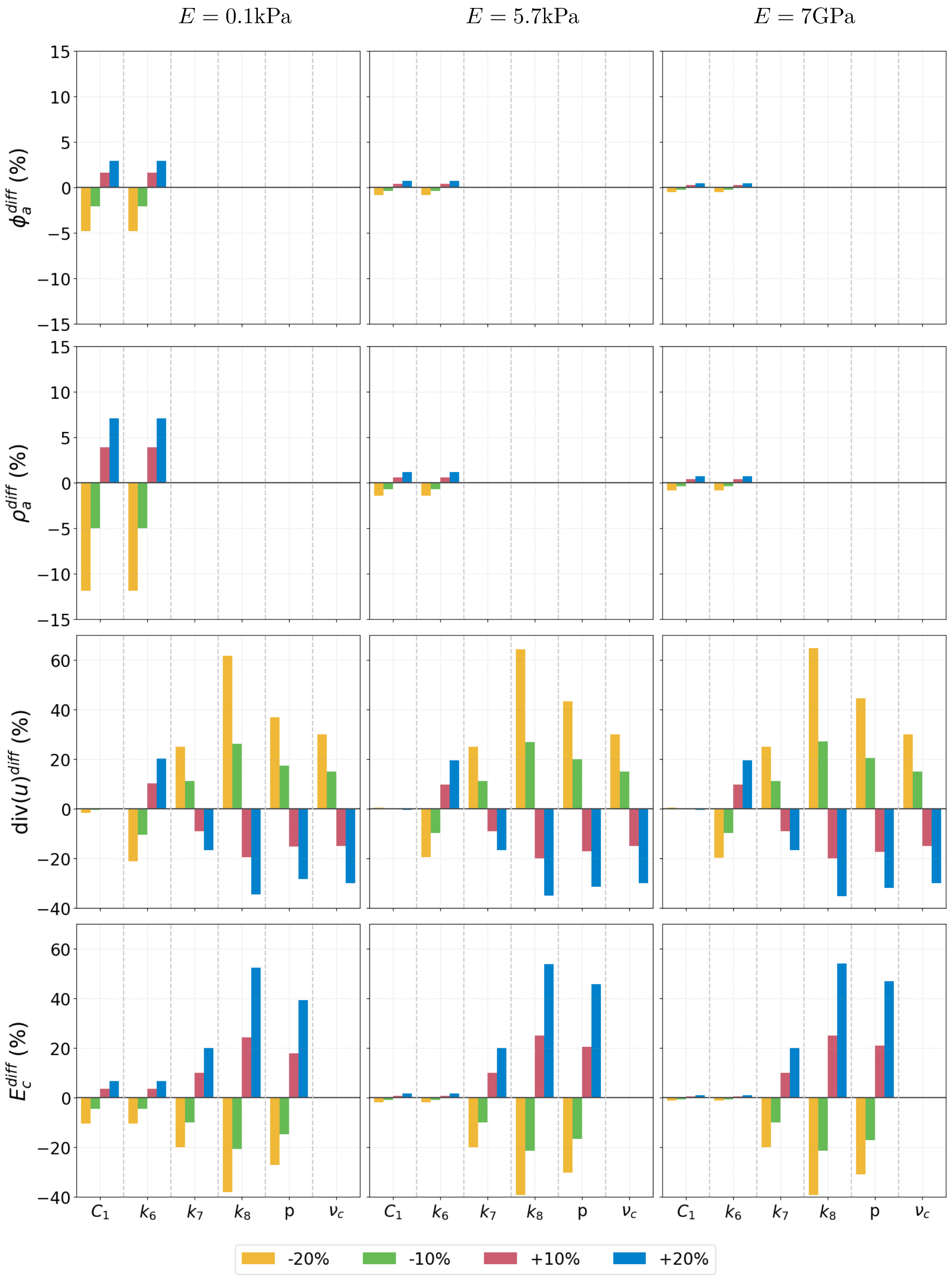

Fig A8. Parameter sensitivity analysis results showing the percentage change in the means of $f(\phi_a)$, $\mathrm{div}(u)$, ϕ_a and ρ_a (%) as response to a percentage change in the parameters C_1, k_6, k_7, k_8, p and ν_c. Here, the percentage change in the mean is defined as $v^{diff} = \frac{\int_\Omega v\mathrm{d}x - \int_\Omega v^*\mathrm{d}x}{\int_\Omega v^*\mathrm{d}x} \cdot 100$, where v the new result and v^* the original result for parameters in Table 1. These results are for the model in Eqs (3), (4), and (6), for the axisymmetric shape and in the case of the 3D stimulus at $T = 100$ s by which time the results are at a steady state. The columns represent different values for the substrate stiffness E and the rows represent the percentage change in the mean of the variables ϕ_a, ρ_a, $\mathrm{div}(u)$ and $E_c = f(\phi_a)$. If not otherwise specified, parameter values are as in Table 1. The corresponding simulation results can be found in Fig 7.

A.7 Simulations with linear viscoelasticity

To model a linear viscoelastic material, we consider the model in Eqs (3) and (6) with the stress defined as

$$\sigma(u) = \lambda(\phi_a)(\nabla \cdot u + \theta_\lambda \nabla \cdot \partial_t u)I + 2\mu(\phi_a)\left(\left(\nabla u + (\nabla u)^T\right) + \theta_\mu\left(\nabla \partial_t u + (\nabla \partial_t u)^T\right)\right), \tag{A8}$$

with the initial condition $u(0, x) = 0$ for $x \in Y$, where θ_λ and θ_μ are the characteristic retardation times. We use a backward Euler discretization such that

$$\begin{aligned}&\left\langle \lambda(\Phi_a^{n-1})\left(1 + \Delta t^{-1}\theta_\lambda\right)(\nabla \cdot U^n)I + 2\mu(\Phi_a^{n-1})(1 + \Delta t^{-1}\theta_\mu)\epsilon(U^n), \epsilon(V)\right\rangle_{Y_h} \\ &\quad = \Delta t^{-1}\left\langle \lambda(\Phi_a^{n-1})\theta_\lambda(\nabla \cdot U^{n-1})I + 2\mu(\Phi_a^{n-1})\theta_\mu \epsilon(U^{n-1}), \epsilon(V)\right\rangle_{Y_h} + \left\langle k_6 \mathbb{P}(P_a^{n-1}\hat{\nu}), V\right\rangle_{\Gamma_h}.\end{aligned}$$

The numerical simulation results are presented in Fig A9.

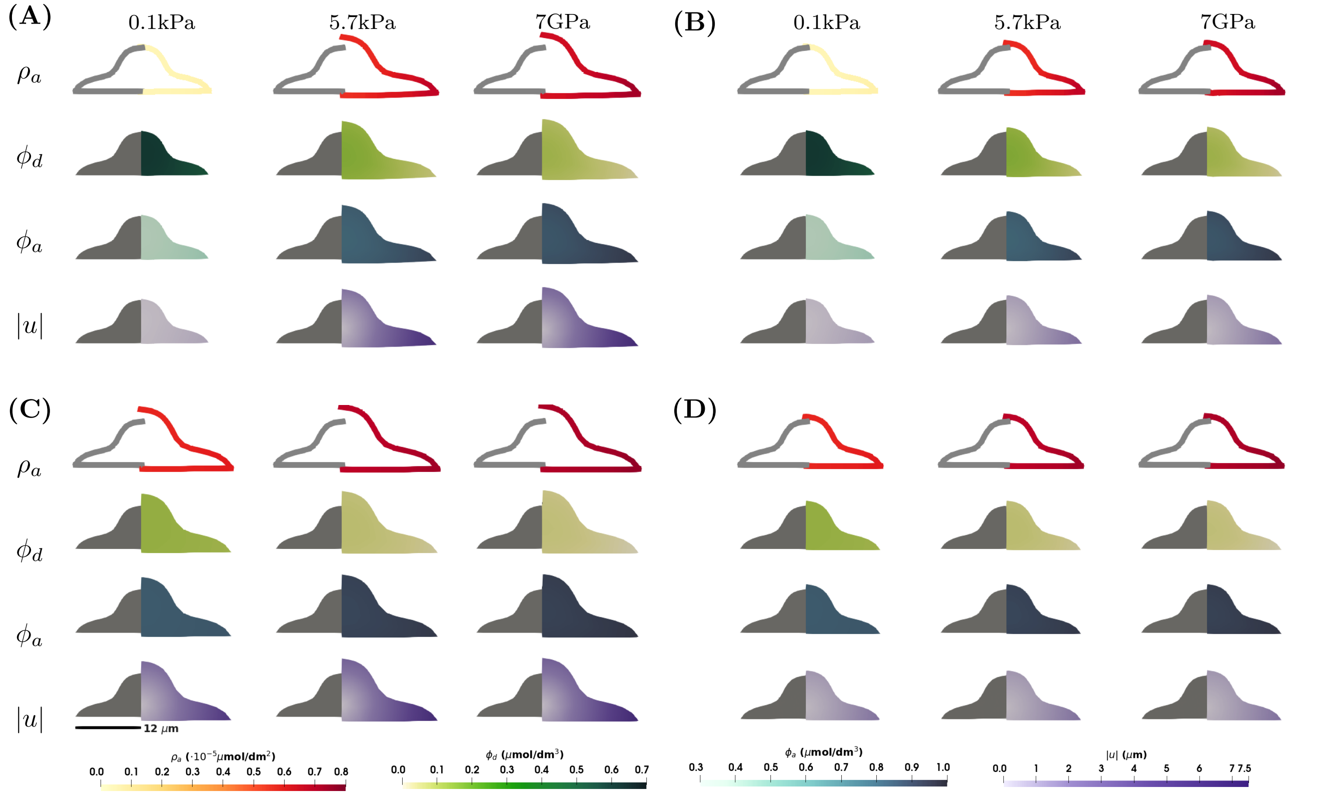

Fig A9. Numerical simulation results showing ρ_a, ϕ_d, ϕ_a and $|u|$ for model (3), (4), (6), and (A8) for the axisymmetric shape and in the case of the 3D stimulus at a steady state at $T = 100$ s. Four different scenarios are considered: **(A)** $C_1 = 0$ (kPa s)$^{-1}$ ($\sigma \not\to \phi_a$) and $E_c = 0.6$ kPa ($\phi_a \not\to E_c$); **(B)** $C_1 = 0$ (kPa s)$^{-1}$ ($\sigma \not\to \phi_a$) and $E_c = f(\phi_a)$ ($\phi_a \to E_c$); **(C)** $C_1 = 0.1$ (kPa s)$^{-1}$ ($\sigma \to \phi_a$) and $E_c = 0.6$ kPa ($\phi_a \not\to E_c$); **(D)** $C_1 = 0.1$ (kPa s)$^{-1}$ ($\sigma \to \phi_a$) and $E_c = f(\phi_a)$ ($\phi_a \to E_c$). Within each subfigure, the rows represent ρ_a, ϕ_d, ϕ_a and $|u|$ on a cross-section of the plane $x_1 = 0$ of the axisymmetric cell, and the columns represent $E = 0.1, 5.7, 7 \cdot 10^6$ kPa. Parameter values as in Table 1, and $\theta_\lambda = \theta_\mu = 1$. The corresponding results with only elastic stress can be found in Fig 7.

References

1. Scott KE, Fraley SI, Rangamani P. A spatial model of YAP/TAZ signaling reveals how stiffness, dimensionality, and shape contribute to emergent outcomes. Proceedings of the National Academy of Sciences. 2021;118(20):e2021571118. doi:10.1073/pnas.2021571118.

2. Le Dévédec SE, Geverts B, de Bont H, Yan K, Verbeek FJ, Houtsmuller AB, et al. The residence time of focal adhesion kinase (FAK) and paxillin at focal adhesions in renal epithelial cells is determined by adhesion size, strength and life cycle status. Journal of Cell Science. 2012;125:4498–4506. doi:10.1242/jcs.104273.

3. Logg A, Mardal KA, Wells G, editors. Automated Solution of Differential Equations by the Finite Element Method. vol. 84 of Lecture Notes in Computational Science and Engineering. Berlin, Heidelberg: Springer Berlin Heidelberg; 2012.

4. Geuzaine C, Remacle JF. Gmsh: A 3-D finite element mesh generator with built-in pre- and post-processing facilities. International Journal for Numerical Methods in Engineering. 2009;79(11):1309–1331. doi:10.1002/nme.2579.

5. Lakkis O, Madzvamuse A, Venkataraman C. Implicit-explicit timestepping with finite element approximation of reaction-diffusion systems on evolving domains. SIAM Journal on Numerical Analysis. 2013;51(4):2309–2330. doi:10.1137/120880112.
