# Supplementary figures and images for: Mathematical modelling of mechanotransduction via RhoA signalling pathways
Source: PLoS Comput Biol. 2025 Jul 31;21(7):e1013305. doi: 10.1371/journal.pcbi.1013305 (PMC12327677; doi:10.1371/journal.pcbi.1013305)

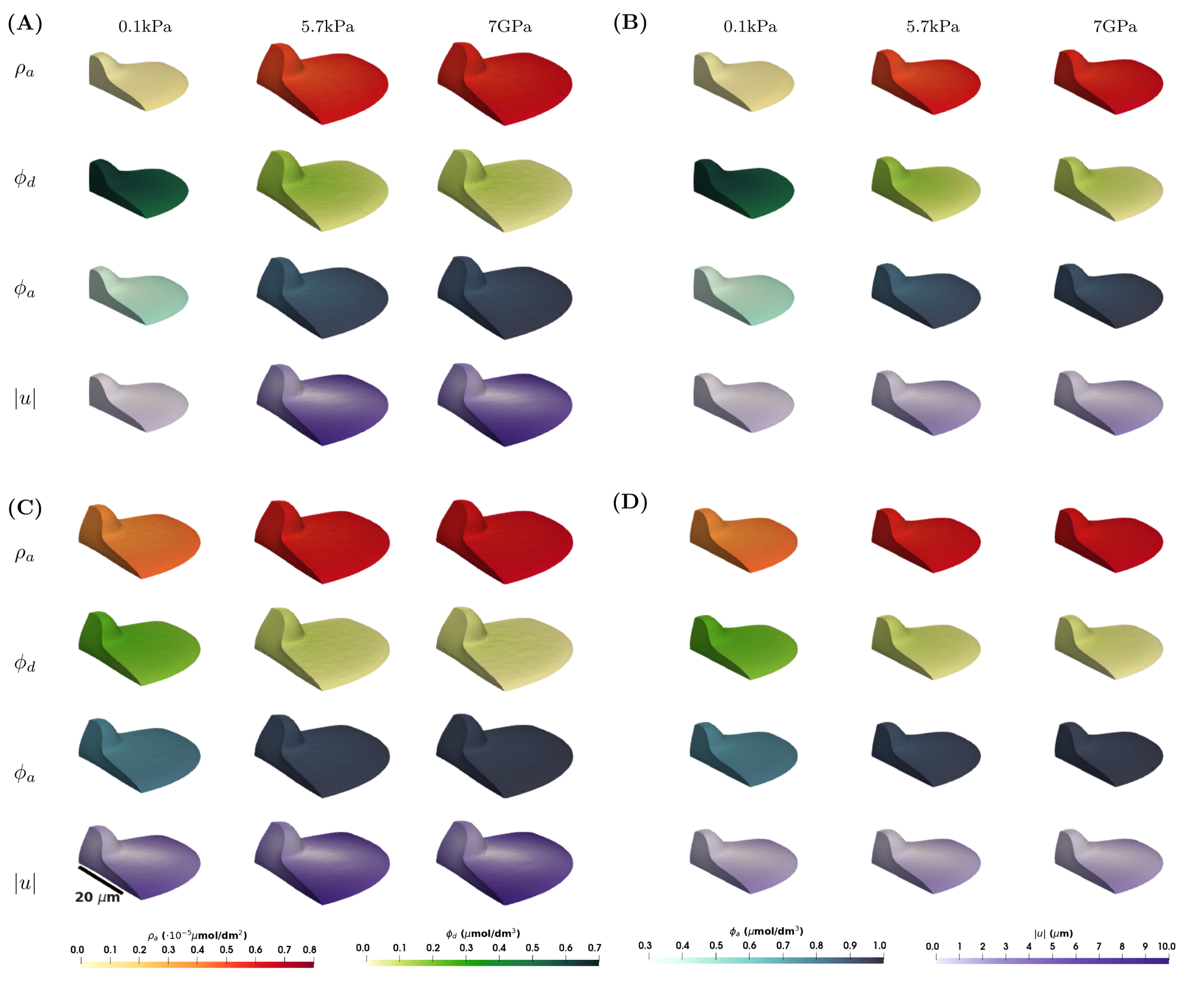

Supplement: S1 Fig — Four different scenarios are considered: (A) C1 = 0 (kPa s)−1 (σ↛ϕa) and Ec = 0.6 kPa (ϕa↛Ec); (B) C1 = 0 (kPa s)−1 (σ↛ϕa) and Ec=f(ϕa) (ϕa→Ec); (C) C1 = 0.1 (kPa s)−1 (σ→ϕa) and Ec = 0.6 kPa (ϕa↛Ec); (D) C1 = 0.1 (kPa s)−1 (σ→ϕa) and Ec=f(ϕa) (ϕa→Ec). Within each subfigure, the rows represent ρa, ϕd, ϕa and |u| on the surface of the cell, and the columns represent E=0.1,5.7,7·106 kPa. Parameter values as in Table 1. (TIF) [file pcbi.1013305.s002.tif]

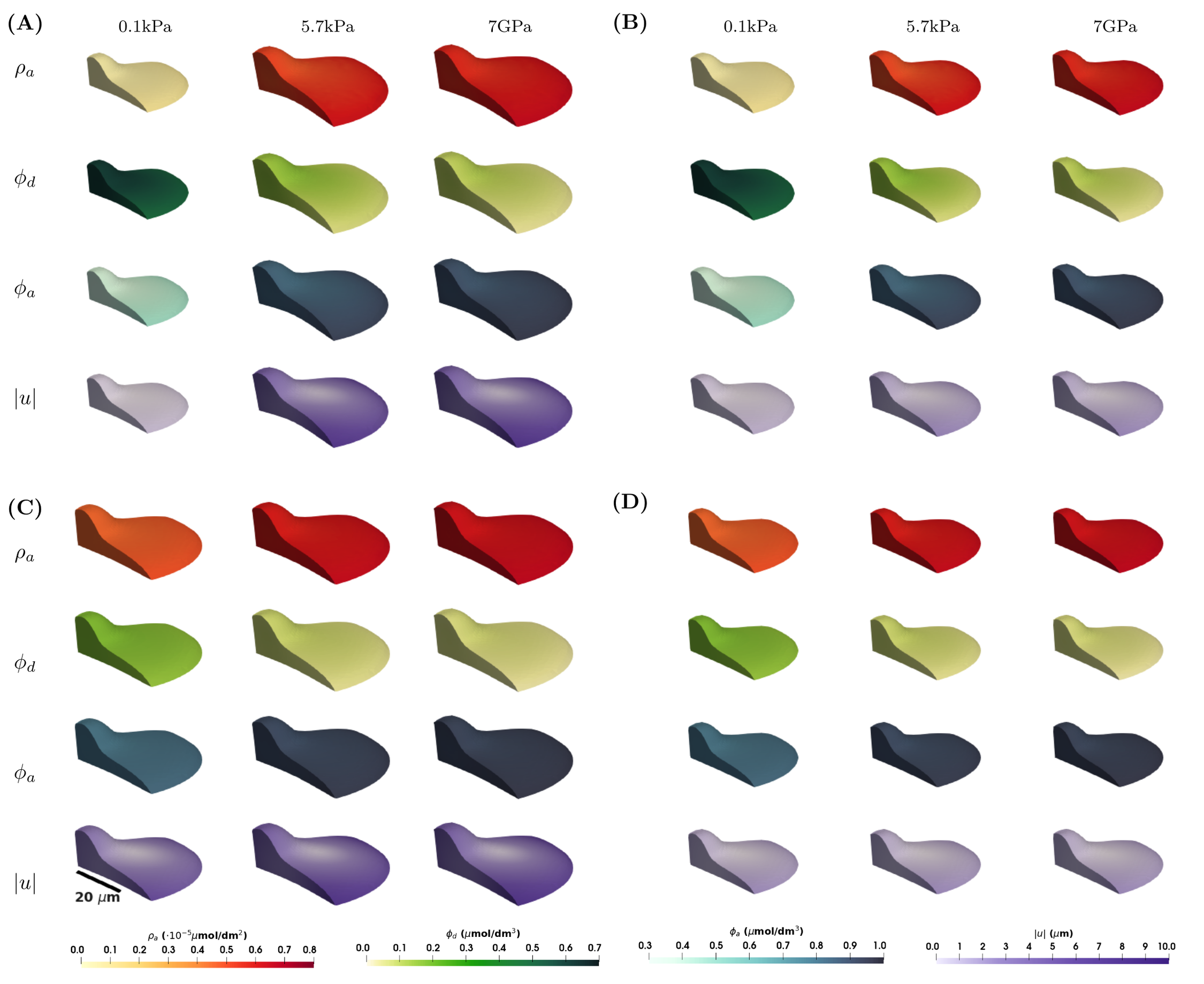

Supplement: S2 Fig — Four different scenarios are considered: (A) C1 = 0 (kPa s)−1 (σ↛ϕa) and Ec = 0.6 kPa (ϕa↛Ec); (B) C1 = 0 (kPa s)−1 (σ↛ϕa) and Ec=f(ϕa) (ϕa→Ec); (C) C1 = 0.1 (kPa s)−1 (σ→ϕa) and Ec = 0.6 kPa (ϕa↛Ec); (D) C1 = 0.1 (kPa s)−1 (σ→ϕa) and Ec=f(ϕa) (ϕa→Ec). Within each subfigure, the rows represent ρa, ϕd, ϕa and |u| on the surface of the cell, and the columns represent E=0.1,5.7,7·106 kPa. Parameter values as in Table 1. (TIF) [file pcbi.1013305.s003.tif]

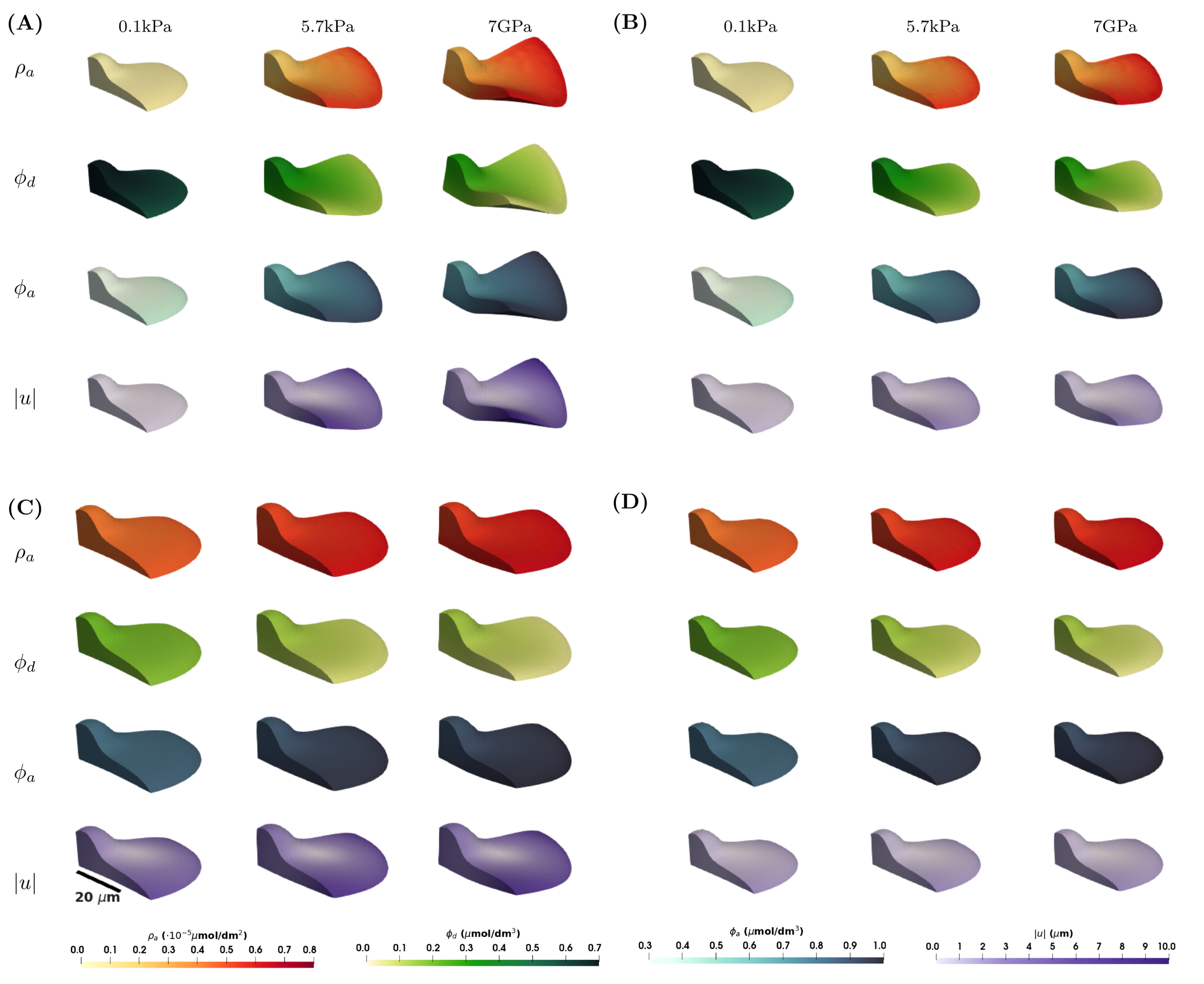

Supplement: S3 Fig — Four different scenarios are considered: (A) C1 = 0 (kPa s)−1 (σ↛ϕa) and Ec = 0.6 kPa (ϕa↛Ec); (B) C1 = 0 (kPa s)−1 (σ↛ϕa) and Ec=f(ϕa) (ϕa→Ec); (C) C1 = 0.1 (kPa s)−1 (σ→ϕa) and Ec = 0.6 kPa (ϕa↛Ec); (D) C1 = 0.1 (kPa s)−1 (σ→ϕa) and Ec=f(ϕa) (ϕa→Ec). Within each subfigure, the rows represent ρa, ϕd, ϕa and |u| on the surface of the cell, and the columns represent E=0.1,5.7,7·106 kPa. Parameter values as in Table 1. (TIF) [file pcbi.1013305.s004.tif]

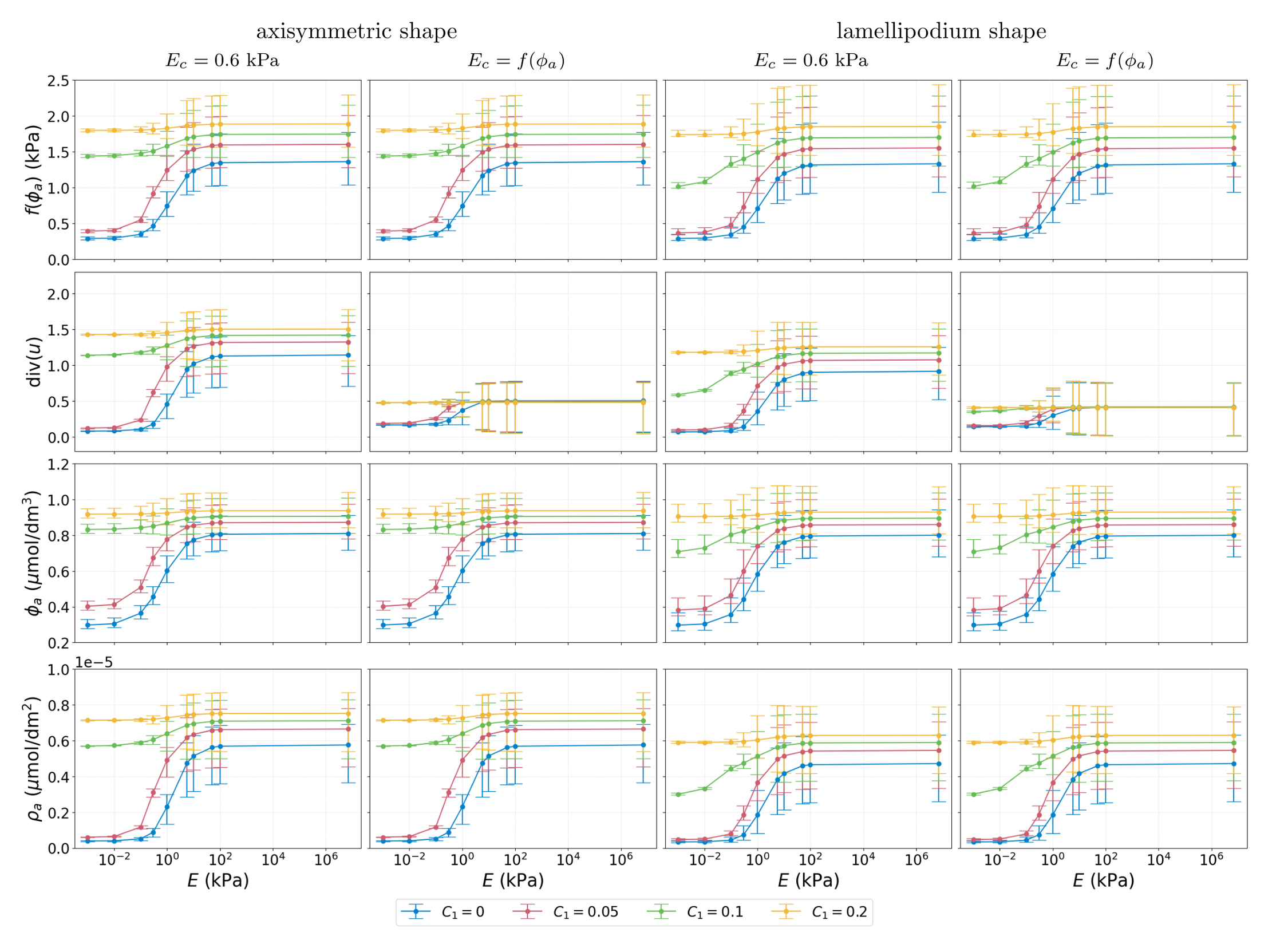

Supplement: S4 Fig — We consider different couplings, four different values for C1, and two different shapes at T = 100 s by which time the results are at a steady state. All other parameter values as in Table 1. (TIF) [file pcbi.1013305.s005.tif]
