# Supplementary material for: Mathematical modelling of mechanotransduction via RhoA signalling pathways
Source: PLoS Comput Biol. 2025 Jul 31;21(7):e1013305. doi: 10.1371/journal.pcbi.1013305 (PMC12327677; doi:10.1371/journal.pcbi.1013305)
Supplement: Attachment — Submitted filename: Response to Reviewers.pdf [file pcbi.1013305.s006.pdf]

Sofie Verhees
Maxwell Institute for Mathematical Sciences
Department of Mathematics
Heriot-Watt University
Edinburgh EH14 4AS, UK

30th April 2025

To: **Dr. Jochen Hub & Prof. Jason Papin**
Academic Editor & Editor-in-Chief
PLOS Computational Biology

Dr. Jochen Hub & Prof. Jason Papin,

Thank you for inviting us to submit a revised manuscript to "PLOS Computational Biology" entitled

"Mathematical modelling of mechanotransduction via RhoA signalling pathways"

by Sofie Verhees, Chandrasekhar Venkatarman, Mariya Ptashnyk.

We appreciate the time and effort you and each of the reviewers have dedicated to providing constructive feedback to strengthen our paper. We have revised the manuscript, incorporated changes that reflect the detailed suggestions, and responded to each comment of the reviewers. We hope that our edits and the responses we provide below satisfactorily address all the issues and concerns you and the reviewers have noted.

In line with the submission guidelines, we note that we have included the Methods section before the Results section, because the Methods section contains crucial explanation on the model and assumptions necessary to understand the numerical simulations in the Results section.

Yours sincerely,

Sofie Verhees, Chandrasekhar Venkataraman, and Mariya Ptashnyk

Responses to the Editor and Reviewers' comments

Editor Comments:

1. *The main manuscript contains an exceptionally large number of figures (20). Please consider whether key results can be presented more concisely in the main text and whether control simulations can be moved to the Supporting Information.*

   We appreciate this comment and agree that focussing on some key results would make the main text more concise and stronger. In response to comment 6 from Reviewer #2, we have already merged Fig 1-2, 3-4, 6-7, 8-9, 11-12, 13-14, 16-17, 18-19 from the original manuscript. Additionally we chose to move Figs 8-9, 13-14, 18-19, and 20 to the Supporting Information, reducing the number of figures to 8.

Reviewer #1 comments:

1. *The authors assume that the cell follows a linear elasticity model, whereas many studies suggest that biological cells exhibit viscoelastic or poroelastic properties, especially over long time scales. It is recommended to discuss the validity of this assumption and consider incorporating viscoelastic effects to improve the biological realism of the model.*

   We thank the reviewer for the recommendation. A viscoelastic or poroelastic model for the mechanics may indeed be more representative of a biological cell. We do note however, that models employing a linear elastic constituive law have been used in previous works and have been effective in reproducing experimental observations (line 110-120 in the revised manuscript). Our focus is modelling a bidirectional coupling between cell stiffness and signalling processes, a minimal model assuming a linear elastic law for the cell mechanics is therefore sufficient for this work, as it avoids unnecessary complications that arise from the consideration of viscous stresses. To demonstrate that we expect our results remain relevant under more complex mechanical models, we have considered the inclusion of viscoelastic effects in the Appendix (Section A.7) and simulation results are almost indistinguishable from an only elastic model.

2. *The model parameters (e.g., RhoA reaction rates, diffusion coefficients, substrate stiffness) are not well justified. The authors should provide references to experimental studies or parameter sensitivity analyses to strengthen the biological basis of their choices.*

   We appreciate the reviewer's concerns regarding the model parameters. We have added references and justification where possible and explored the parameter sensitivity of the model in the Supporting Information, where we show the percentage change in the means of $f(\phi_a)$, $\text{div}(u)$, ϕ_a, and ρ_a under a percentage change of -20%, -10%, 10%, and 20% of all new parameters introduced in the paper.

3. *RhoA primarily regulates actin cytoskeleton remodeling, influencing cellular contractility and adhesion dynamics. However, the current model does not explicitly incorporate F-actin dynamics. Computational studies on cytoskeletal remodeling have been extensively explored in the literature, and these sources must be acknowledged and integrated into the manuscript. The authors may refer to the following studies:*

   - *Predicting YAP/TAZ nuclear translocation in response to ECM mechanosensing.*
   - *Directed cell migration towards softer environments.*

   We thank the reviewer for pointing out these interesting studies. We have referred to them where appropriate. We have also clarified that F-actin is downstream from activated FAK and, as apparent from the results in (Scott et al., 2021), we can use activated FAK as a proxy for F-actin (line 130-134 in revised manuscript).

Reviewer #2 comments:

1. *It is stated that "inactive species are assumed to be cytoplasm resident and activated forms membrane resident". However, while it appears that the latter actually evolves on the membrane Gamma, FAK seems to live in the cytoplasm in both forms.*

   We appreciate the reviewer pointing out this confusion. FAK indeed lives in the cytoplasm in both forms and we have rephrased this sentence to "FAK, both active and inactive, is assumed to be cytoplasm resident and activated RhoA membrane resident" to more accurately represent the model.

2. *This confusion transfers to the figures, where it is not clear what is shown. For instance, in the 2D case, is Fig.1 showing a section of the cell, or the membrane with an axisymmetric geometry? And then what about Fig.2? And so on ....*

   We appreciate the reviewer's comment and have added in the caption of the figures if the simulations show a cross-section or a surface to more clearly describe what is portrayed regarding the cell shape.

3. *The fact that an evolution equation depends on an initial value is odd (say last equation in (1)). Since the total mass of rhoA seems to be conserved, it is better to refer to such a quantity, that is related to* $\rho_d^0 + \rho_a^0/n_r$.

   We thank the reviewer for this comment and the suggestion. We have introduced the total mass of RhoA, $M_\rho = \int_Y \rho_d^0 \, \mathrm{d}x + \int_\Gamma \rho_a^0 \, \mathrm{d}s$, and replaced this accordingly in Eq(1) and Table 1.

4. *A big problem regards the dimensions of the quantities introduced. This makes unclear what are the parameters actually used. For instance,*
   We thank the reviewer for their detailed comments. We have addressed the concerns regarding the dimension of the parameters in the changes as described here...

   4.1. *rhoA and phi are said to be concentrations, but the former is measured in* $\#/m^2$ *and the latter in moles (which is not a concentration)*

   We agree that different dimensions for RhoA and FAK can lead to confusion. Our choice for dimension of RhoA was based on being able to compare results to previous works, but we recognize the confusion this caused. We have changed the dimension of RhoA to μmol/dm^2 in all figures and text, and only convert to $\#/m^2$ when the comparison with previous work is made in the Appendix. We recognize that this disparity might have created a lack of clarity to the dimensions of FAK, which is measured in μM (micromolar), but hope that changing the notation of the dimension of FAK (from μM to μmol/dm^3) to match RhoA (μmol/dm^2) has resolved this.

   4.2. *In Eq.(2) the dimensions of* k_8 *will depend on the power p, as well as the dimensions of* k_7 *in order to eventually have an* E_c *that is measured in Pascal. Certainly* k_7 *and* k_8 *are not* s^{-1} *as stated in Table 2. However, it would be better to define* E_c *(that I believe is then called f) as* $E_c = k_7(1 + (k_8\phi_a)^p)$ *where the dimensions of* k_8 *are the inverse of those of* ϕ_a

   We thank the reviewer for catching this inaccuracy in the dimensions of k_7 and k_8, and their suggestion for redefining E_c. We have adapted the definition of E_c and the dimension of the parameters in Table 1b (previously Table 2) as suggested.

   4.3. C_1 *seems to be a pure number but its dimensions are 1/(Pa s). Then being either 0 or 1 like an on-off switch makes little sense. There must be a parameter measuring the stress-induced activation of FAK.*

   We agree that C_1 is a parameter measuring the stress-induced activated of FAK. We have

redefined this parameter in Table 1 to make this more clear and corrected its dimension. In Fig 5, 10, 15, 10 (original manuscript) we explore different values of C_1 and their effect on the results.

5. *The presentation of the figures can be improved. In addition to the confusion mentioned above, I suggest to replace C=0 and C=1 with a more descriptive notation, such as* $\sigma \not\to FAK$ *and* $\sigma \to FAK$*. Similarly, I would use* $FAK \not\to E$ *and* $FAK \to E$ *for the columns. Then, does* $E_c = 0.6$ *corresponds to the parameters used for* f *when the effect of FAK on the Young modulus is switched off?*

   We thank the reviewer for this comment and their suggestions. We agree that a more intuitive description could be helpful to explain the figures, and have added those in the figures as suggested. We also recognize that the reader might appreciate how the intuition relates to the parameters in the model, and have therefore included both in the figures.

6. *In addition, graphically speaking, while one figure presents both concentrations (why not* ϕ_d*?) the other presents the deformation only (with some confusion induced by a reordering of the "table" of cases). Would it be possible to use the same format for all variables? Or put phi, rho, and u for instance one below the other for the different cases? Or split in three figures one per state variable?*

   We appreciate the reviewer commenting on the clarity of the figure formats. We like the suggestion of including ϕ_d and have added this to the results. Secondly, to have a more unified approach to presenting the results, we have projected the concentrations of FAK and RhoA on the deformed domain, so all variables can be included in one figure on the deformed domain.

7. *The so-called lamellipodium configuration is not clear starting from its introduction at line 177. In addition, the results of the simulations give an axisymmetric distribution of the concentrations and deformations that are not axisymmetric. Why is that? Are the Authors sure they are using the correct boundary conditions on the sector they study?*

   We thank the reviewer for this comment. The shape has been introduced in the previous section (line 161-165 in original manuscript/ line 171-175 in revised manuscript) and we recall this fact when presenting simulations. The lamellipodium configuration is not axisymmetric and we therefore would not expect an axisymmetric distribution of the concentrations and deformations. We have addressed this confusion by adding clarification when discussing the results of the lamellipodium shape. We also hope by specifying in the caption if the cell is presented as a cross-section or as the surface will help clarify this concern.

8. *in Eq.(5),* $\cdot$ *is used to mean different operations, a scalar product between two vectors and the multiplication of a matrix (the stress) by a vector (the normal). The following sentence is a bit obscure as it is written. After all,* Π_τ *is just the shear stress at the surface.*

   We appreciate this comment and agree that Eq.(5) was not clear as stated. We have removed $\cdot$ to represent the multiplication of a matrix and a vector.
