# Supplementary material for: Mathematical modelling of mechanotransduction via RhoA signalling pathways
Source: PLoS Comput Biol. 2025 Jul 31;21(7):e1013305. doi: 10.1371/journal.pcbi.1013305 (PMC12327677; doi:10.1371/journal.pcbi.1013305)
Supplement: Attachment — Submitted filename: Response_to_Reviewers_auresp_2.pdf [file pcbi.1013305.s007.pdf]

Sofie Verhees
Maxwell Institute for Mathematical Sciences
Department of Mathematics
Heriot-Watt University
Edinburgh EH14 4AS, UK

7th July 2025

To: **Dr. Jochen Hub & Dr. Feilim Mac Gabhann**
Academic Editor & Editor-in-Chief
PLOS Computational Biology

Dr. Jochen Hub & Dr. Feilim Mac Gabhann,

Thank you for inviting us to submit a revised manuscript to "PLOS Computational Biology" entitled

"Mathematical modelling of mechanotransduction via RhoA signalling pathways"

by Sofie Verhees, Chandrasekhar Venkatarman, Mariya Ptashnyk.

We thank the reviewers for their time and effort in reviewing our paper. We have revised the manuscript based on the suggestions, see below for a detailed response.

Yours sincerely,

Sofie Verhees, Chandrasekhar Venkataraman, and Mariya Ptashnyk

Responses to the Editor and Reviewers' comments

Reviewer #2 comments:

1. *What about insert a sketch of the protein relations and their location at the beginning of the modeling section?*

   We appreciate this suggestion and have added a sketch of the couplings represented in the model at the start of the methods section (Fig 1).

2. *What about recalling that (5) represents a shear stress-free condition?*

   We appreciate the reviewer's comment and have added extra explanation in line 147-148 recalling that (5) represents a shear stress free condition.
